# Sucrosomial® iron effectiveness in recovering from mild and moderate iron-deficiency anemia in the postpartum period

**DOI:** 10.1186/s12884-023-05658-7

**Published:** 2023-05-17

**Authors:** Edu Antoine, Claudia Mehedintu, Mihai Mitran, Doru Diculescu

**Affiliations:** 1 Department of Obstetrics-Gynecology, Clinical Hospital “Nicolae Malaxa”, Vergului 12 Road, 022441 Bucharest, Romania; 2 Department of Obstetrics-Gynecology, Clinical Hospital “Panait Sirbu”, Bucharest, Romania; 3 Department of Obstetrics-Gynecology, Clinical Hospital “Dominic Stanca”, Cluj, Romania

**Keywords:** Postpartum, Iron deficiency, Sucrosomial® iron, Women, Mild anaemia, Moderate anaemia

## Abstract

**Background:**

Postpartum iron deficiency anemia (PPIDA) is highly prevalent in developing countries where it constitutes an important cause of maternal morbidity and mortality. Potential determinants of PPIDA are prepartum iron deficiency or iron deficiency anemia in association with severe blood loss during delivery. We investigated the efficacy of oral Sucrosomial® iron for recover from mild-to-moderate PPIDA.

**Methods:**

This pilot study was conducted in three medical centers in Romania.

Adult women (≥ 18y) with mild (hemoglobin [Hb] 9–11 g/dL) or moderate (Hb 7–9 g/dL) PPIDA diagnosed at screening (2–24 h after delivery) were eligible.

Women with mild PPIDA received oral Sucrosomial® iron (Pharmanutra, S.p.A, Italy) once daily (30 mg elemental iron per capsule) for 60 days. Those with moderate PPIDA received oral Sucrosomial® iron twice daily (60 mg elemental iron) for 10 days, followed by a 50-day course of oral Sucrosomial® iron once daily (30 mg elemental iron). Laboratory parameters, as well as subjective clinical symptoms using a 3-point Likert Scale, were assessed at baseline and on study days 10, 30 and 60.

**Results:**

Sixty anemic women entered the study, but three were missed during follow-up. At day 60, a Hb rise was observed in both groups (+ 3.6 ± 1.5 g/dL; *p* < 0.01), 81% experienced correction of anemia (Hb ≥ 12 g/dL), 36% achieved a ferritin concentration ≥ 30 ng/mL (*p* < 0.05), and 54% a transferrin saturation (TSAT) ≥ 20% (*p* < 0.01). For women still anemic at day 60, mean Hb was close to normality (11.3 ± 0.8 g/dL). Resolution of IDA-associated clinical symptoms was already observed just 10 days after treatment initiation. No patient discontinued treatment due to gastrointestinal adverse events.

**Conclusions:**

Sucrosomial® iron was shown to be potentially effective and well tolerated at treating mild and moderate PPIDA. These results encourage the use of oral Sucrosomial® iron as a treatment option for PPIDA, but larger studies with longer follow-up are warrant.

## Background

In the postpartum period, both iron deficiency (PPID) and iron deficiency anemia (PPIDA) are common and represent significant health problems in women of reproductive age [1]. In a series of 43,807 women who gave birth between 1993 and 2008 in Germany, the prevalence of PPA (hemoglobin [Hb] < 10 g/dL) 24–48 h after delivery was 22%. This prevalence increased up to 50% when PPA was defined as Hb < 11 g/dL [2]. In developing countries, the prevalence of PPA is in the range of 50–80% and, together with postpartum hemorrhage represents a major cause of maternal morbidity and mortality [1].

PPIDA leads to prolonged hospital stay, impaired quality of life, reduced cognitive abilities, emotional instability, and depression [3–5]. In addition to anemia, ID has other manifestations, such as tiredness, hair loss, and restless legs [6]. However, the importance of PPIDA seems to be overlooked by both obstetricians and patients, even after severe postpartum hemorrhage [1, 7].

Therefore, the most frequent predisposing factors (placenta previa [OR 4.7], anemia during pregnancy [OR 2.7], multiple birth [OR 2.2], and blood loss > 1000 mL [OR 74.7]) [2] should be identified and modified when possible. Additionally, routine PPIDA screening should be considered, especially in women with antenatal ID/IDA and/or significant peripartum bleeding, in order to provide early and appropriate treatment [1].

As for women with mild-to-moderate anaemia postpartum who are haemodynamically stable, asymptomatic, or mildly symptomatic, daily administration of oral ferrous iron salts for three months has been recommend [1]. However, absorption and tolerance should be taken into account: 1) Up to 50–70% of patients on oral iron sulfate experience gastrointestinal side effects due to exposure to unabsorbed iron; 2) Co-administration of oral iron with food or drugs (e.g., antacids, proton pump inhibitors) may drastically reduce iron absorption; 3) In addition, hepcidin is up-regulated when a high iron dose is ingested, which may reduce the absorption of the next oral iron dose [8]. Thus, whenever possible, Hb concentration should be determined after 2–4 weeks of treatment initiation. For those with confirmed ID and lack of responsiveness or intolerance to oral iron, intravenous treatment was the recommended treatment alternative [1].

Sucrosomial® iron (SI, Pharmanutra, S.p.A., Italy) is a newer oral iron formulation consisting of a ferric pyrophosphate covered by phospholipids and sucrester matrix. The absorption of SI is mostly hepcidin-independent, as is taken up in the lymphatic circulation through transcellular and M cell-mediated endocytosis routes [9]. Oral SI has been shown to be safe and efficacious for treating ID and IDA in a variety of clinical scenarios [9].

As oral SI has been shown efficacious and well tolerated in the prophylaxis of pregnancy-associated ID and IDA [10], and preliminary data suggested its efficacy in PPIDA [11], this non-comparative pilot study aimed to investigate whether supplementation with SI was efficacious at correcting mild or moderate PPIDA (as measured by significant improvement in hemoglobin levels over time for each group when compared to baseline levels; primary outcome) and improving PPIDA-associated subjective symptoms (as measured by a 3-point Likert scale; secondary outcome).

## Methods

### Study design and participants

This was a multicenter pilot study conducted at three obstetrics facilities (both public and private) in two Romanian cities (Bucharest and Cluj-Napoca) from October 2018 to June 2020. Women were screened for PPIDA (Hb < 11 g/dL, ferritin < 12 ng/mL, transferrin saturation < 20%) 2–24 h after vaginal delivery (T0, baseline). Those presenting with confirmed mild (Hb = 9–11 g/dL) or moderate (Hb = 7–9 g/dL) PPIDA at screening and providing informed written consent were recruited to participate in the study. Study participants were allocated to the corresponding treatment regime (see below), and followed up for 60 days. The study was approved by the National Bioethics Committee for Medicines and Medical Devices under the protocol number 27S/1–4/15.10.2018.

The manufacturer of SI (Pharmanutra, S.p.A., Italy) financed the study.

### Intervention and follow-up

Consecutive women with mild PPIDA (Group A) were treated with one daily Sucrosomial® iron capsule (30 mg elemental iron/day) for 60 days. Those with moderate PPIDA (Group B) received one capsule of Sucrosomial® iron twice daily (60 mg elemental iron/day) for 10 days, followed by a 50-day course of one daily Sucrosomial® iron capsule (30 mg elemental iron/day).

Treatment compliance, laboratory parameters, and subjective clinical symptoms assessment, were completed by the attending physician at baseline (T0) and at days 10 (T10), 30 (T30) and 60 (T60) of the treatment period.

### Data collection

At each visit, laboratory parameters were evaluated to assess anemia and iron status. The normal ranges at our laboratory included: Hb (12.1–15.1 g/dL), ferritin (13–150 ng/mL), transferrin saturation (TSAT, 16–45%), reticulocyte counts (Retic, 30–120 × 103/μL), and C-reactive protein (CRP, < 0.5 mg/dL).

The severity of possible subjective symptoms associated with PPIDA was assessed on a 3-point Likert Scale (Severe/Mild/Not present or Low-Poor/Good/Excellent), including weakness, tiredness, headache, concentration, appetite, and lower limb pain (due to possible restless legs syndrome) [12]. All evaluations were performed directly by the attending physicians during baseline and follow-up visits through the administration of questionnaires.

### Sample size

With an effect size of 0.5 between the first and mean of the last two Hb measurements, a sample of 27 patients provided 80% statistical power and an alpha error of 0.05. The study sample was increased to 30 patients to account for a 10% loss during follow-up.

### Statistical analysis

The normality of biomarker data distributions was assessed using the Shapiro–Wilk test and Q-Q curves. Normally distributed variables were represented as mean ± standard deviation (m ± SD), while non-normally distributed variables were represented as median and interquartile range [IQ]. Likert-scale variables were represented as percentages. The temporal trends of all variables for each clinical condition (mild anemia, moderate anemia) were analyzed using repeated measure ANOVA and post-hoc comparisons. Specifically, normally distributed biomarkers were analyzed using Fisher’s test and Holm's post-hoc tests, while non-normally distributed biomarkers and Likert-scale variables were analyzed using Friedman’s test and Conover's post-hoc tests. All statistical analyses were conducted using the R statistical software [13], and statistical significance was set at *p* < 0.05.

## Results

**Fig. 1:**
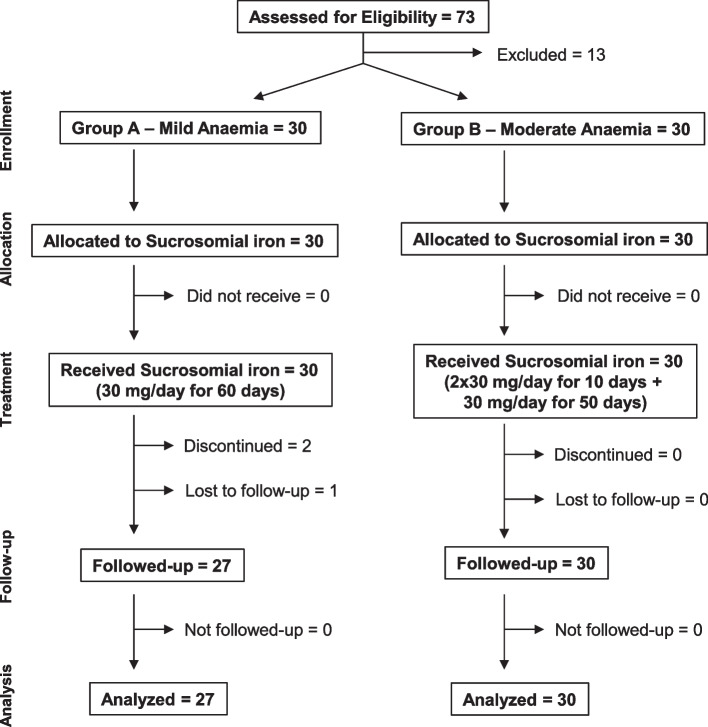
Patients’ disposition in the study

Seventy-three consecutive women presenting with mild or moderate postpartum anemia were approached for recruitment. Thirteen were excluded because they had non-ID anemia. The remaining 60 women (30 with mild anemia, Group A; 30 with moderate anemia, Group B) were enrolled in the study (Fig. 1). As shown in Table 1, there were no differences in demographic and baseline clinical characteristics between the two groups of subjects.

**Table 1:** Demographic and baseline clinical characteristics. Data are presented as mean ± SD, or frequencies

	Group A	Group B	*p*-value
	Mild anemia	Moderate anemia	
	*n* = 27	*n* = 30	
Age (years)	28 ± 6	28 ± 5	0.982
Weight (kg)	71 ± 13	73 ± 13	0.325
Height (cm)	163 ± 6	163 ± 6	0.802
Settlement (rural/urban)	9/21	7/23	0.784
Education (middle school/high school/university)	8/12/10	7/12/11	0.808
Body mass index (kg/m^2^)	27.1 ± 4.1	29.4 ± 7.5	0.146
Obesity (BMI > 30), n (%)	4 (13)	10 (33)	0.132
Systolic blood pressure (mmHg)	112 ± 10	112 ± 10	0.999
Heart rate (bpm)	82 ± 8	85 ± 9	0.153
Body Temperature (ºC)	36.1 ± 0.3	36.1 ± 0.4	0.789
Breathing Rate (n/min)	16 ± 2	16 ± 2	0.756
Hemoglobin (g/dL)	10.1 ± 0.4	8.4 ± 0.6	0.001

Patients’ disposition and follow-up are depicted in Fig. 1. Three patients from group A discontinued iron supplementation or were lost during follow-up. The first one developed infectious mastitis (not related to iron treatment) and declined to continue treatment after T1, the second was advised externally to discontinue treatment before T1, and the third did not attend the follow-up visits. Finally, 57 patients (27 in Group A and 30 in Group B) completed the 60-days follow-up program and were included in the data analysis. No patient discontinued treatment due to gastrointestinal adverse events.

**Fig. 2:**
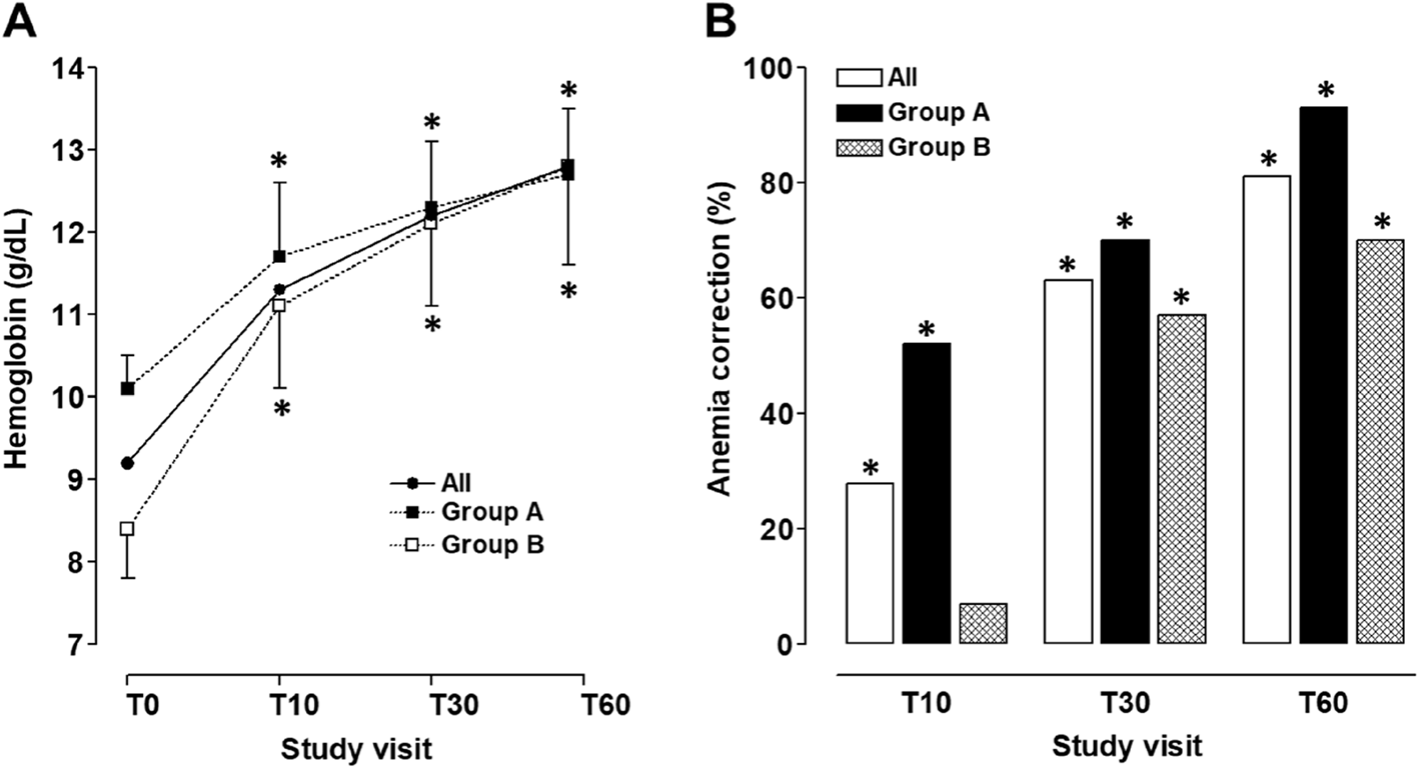
Hemoglobin concentrations (**A**) and percentages of anemia correction (**B**) at the different time-points of the follow-up in all patients (*n* = 57), and patients with mild (Group A, *n* = 27) or moderate (Group B, *n* = 30) PPIDA treated with oral Sucrosomial® iron for 60 days. T0, treatment initiation, T10, T30 and T60, days 10, 30 and 60 after treatment initiation, respectively. *p < 0.01 respect to T0

Iron replacement therapy with oral Sucrosomial® iron led to improvement in laboratory parameters in both study groups (Table 2). For the entire study sample (*n* = 57), Hb concentration increased from 9.2 ± 1.0 g/dL at T0 to 12.8 ± 1.0 at T60 (mean Hb increment: 3.6 ± 1.5 g/dL; *p* < 0.01) (Fig. 2A), and 81% of patients experienced correction of anemia (Hb ≥ 12 g/dL) (Fig. 2B). For women still anemic at day 60, the mean Hb was close to normal (11.3 ± 0.8 g/dL). CRP concentrations abruptly decreases from T0 to T10, remaining low thereafter (Table 2A). There was a progressive increase in the percentage of women attaining a ferritin concentration ≥ 30 ng/mL (*p* < 0.05) and/or a TSAT value ≥ 20% (*p* < 0.01) from T0 to T60 (Table 2A). However, as there were two different iron supplementation schedule according to anemia severity, these changes were analyzed separately for women presenting with mild (Group A) or moderate (Group B) PPIDA.

**Table 2:** Time-course of laboratory parameters in patients with pospartum IDA treated with oral Sucrosomial® iron for 60 days: A, all IDA patients; B, mild IDA (Group A, *n* = 27); C, and moderate IDA (Group B, *n* = 30). ∆Hb, Hb increment from T0 to T10, T30 or T60, respectively. T0, treatment initiation; T10, T30 and T60, days 10, 30 and 60 after treatment initiation, respectively

	**T0**	**T10**	**T30**	**T60**	***p*-value**
**A. All patients (*n* = 57)**					
∆Hb (g/dL)	–-	2.1 ± 0.9	3.1 ± 1.2	3.6 ± 1.5	< 0.01
CRP (mg/dL)	4.9 [2.8 – 7.7]	0.7 [0.4 – 0.7]	0.4 [0.2 – 0.9]	0.2 [0.1 – 0.7]	< 0.01
Ferritin (ng/mL)	21 [12-29]	29[19 – 47]	30[18 – 42]	25 [15-31]	NS
Ferritin ≥ 30 (%)	22	49	49	36	< 0.05
TSAT (%)	11 [8-14]	14 [9-19]	18 [12-25]	18 [12-25]	< 0.01
TSAT ≥ 20 (%)	6	23	42	45	< 0.01
**B. Mild anemia ( Group A; *n* = 27)**					
∆Hb (g/dL)	–-	1.7 ± 0.8	2.4 ± 0.6	2.7 ± 0.9	< 0.01
CRP (mg/dL)	5.2 [2.8 – 9.3]	0.4 [0.2 – 0.7]	0.3 [0.2 – 0.8]	0.2 [0.1 – 0.7]	< 0.01
Ferritin (ng/mL)	20 [15-28]	28[18 – 44]	29 [18 – 45]	28 [15 – 38]	NS
Ferritin ≥ 30 (%)	22	40	44	35	0.381
TSAT (%)	11 [8-14]	15 [9-21]	18 [14-33]	19 [12-27]	< 0.01
TSAT ≥ 20 (%)	12	37	52	54	< 0.01
**C. Moderate anemia (Group B; *n* = 30)**					
∆Hb (g/dL)	–-	2.4 ± 0.9	3.7 ± 1.3	4.4 ± 1.5	< 0.01
CRP (mg/dL)	4.4 [2.9 – 6.3]	1.4 [0.7 – 1.9]	0.5 [0.3 – 0.9]	0.4 [0.1 – 0.7]	< 0.01
Ferritin (ng/ml)	19 [10-28]	31 [26 – 64]	31 [21 – 43]	24 [17-31]	NS
Ferritin ≥ 30 (%)	22	61	55	37	0.520
TSAT (%)	12 [8-15]	12 [9-16]	16 [12-22]	17 [12-21]	< 0.01
TSAT ≥ 20 (%)	0	10	33	42	< 0.01

In Group A, mean Hb concentration at T0 was 10.1 ± 0.4 g/dL. The daily administration of 30 mg elemental iron for 60 days resulted in a mean Hb increment of 2.7 ± 0.9 g/dL from T0 to T60 (*p* < 0.01), leading to normalization of the mean Hb concentration at T60 (12.7 ± 0.8 g/dL; *p* < 0.01) (Fig. 2A). This led to anemia correction in 25 out of 27 women (93%) in this group (Fig. 2B). For the 2 women who were still anemic at T60, their Hb concentrations were close to normal (10.9 and 11.5 g/dL).

There were no significant changes in ferritin concentration throughout the study period or in the percentage of patients achieving a normal ferritin concentration (≥ 30 ng/mL) (Table 2B). However, the TSAT significantly increased throughout the study period from T10 onwards, as did the percentage of patients achieving a TSAT ≥ 20% (Table 2B).

In Group B, the mean Hb concentration at T0 was 8.4 ± 0.6 g/dL. The administration of 60 mg elemental iron/day from T0 to T10, followed by 30 mg/day from T10 to T60, resulted in a mean Hb increment of 4.4 ± 1.5 g/dL from T0 to T60 (*p* < 0.01), leading to normalization of the mean Hb concentration a T60 (12.8 ± 1.2 g/dL; *p* < 0.01) (Fig. 2A). This led to anemia correction in 21 out of 30 women (70%) in this group (Fig. 2B). For the 9 women who were still anemic at T60, the mean Hb concentration was close to normal (11.3 ± 0.8 g/dL).

Again, there were no significant changes in ferritin concentration throughout the study period or in the percentage of patients achieving a normal ferritin concentration (≥ 30 ng/mL) (Table 2C). However, the TSAT significantly increased throughout the study period from T10 onwards, as did the percentage of patients achieving a TSAT ≥ 20% (Table 2C).

**Fig. 3:**
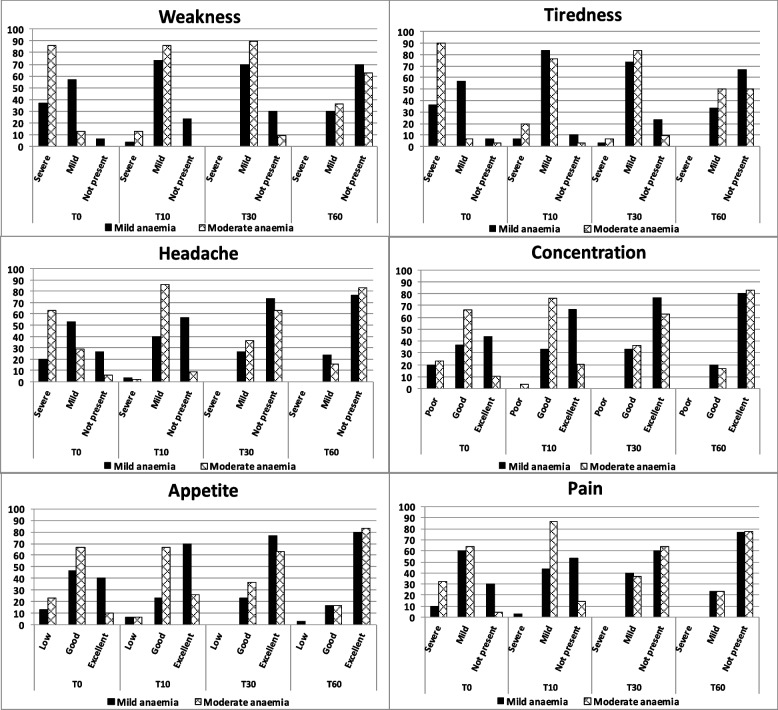
Evolution of clinical symptoms in mild (black bars) and moderate (grey bars) anemia

Remarkably, the beneficial effects of replacement therapy with oral Sucrosomial® iron on both Hb increment (Fig. 2A) and anemia correction (Fig. 2B) were observed as early as 10 day after treatment initiation. This rapid effect of Sucrosomial® iron administration had an impact on clinical symptoms associated with mild and moderate PPIDA. As shown in Fig. 3, there is a clear trend towards improvement in the time-course of all clinical symptoms in both mild IDA (black bars) and moderate IDA (grey bars). From day 10 onwards, there were no clinically relevant symptoms in either group.

## Discussion

Iron supplementation has been proven to be effective in increasing hemoglobin levels in pregnant and postpartum women, and it has been suggested as a public health solution to improve maternal and child health outcomes across different age groups [14, 15].

In this pilot study, we demonstrated that replacement therapy with Sucrosomial® iron, an innovative oral formulation, was well tolerated (no woman discontinued treatment due to gastrointestinal adverse events) and potentially effective in treating mild and moderate PPIDA. Mean Hb increment was 3.6 ± 1.5 g/dL, and 81% of patients achieved Hb levels of ≥ 12 g/dL at the end of the treatment period. These results are comparable to those obtained 6–24 weeks after the administration of intravenous iron to women with moderate PPIDA (mean Hb increment, 3.8 g/dL; anemia correction, 88%; *n* = 658) [16–19].

It has been argued that intravenous iron administration to women with moderate PPIDA results in a faster increase in Hb levels compared to oral iron (ferrous sulfate). However, in moderate IDA, the Hb increment (2.4 ± 0.9 g/dL) observed after 10 days of iron replacement therapy with Sucrosomial® iron (60 mg/day, Hb in) was similar to those observed 2 weeks after intravenous iron administration (2.2 g/dL – 3.2 g/dL) [17, 20–23], which is reflected in the rapid improvement of clinical symptoms (Fig. 3). These effects of Sucrosomial® iron on quality of life have also been observed in heart failure patients with reduced ejection fraction and iron deficiency [24].

As mentioned erlier, Sucrosomial® iron absorption is mostly hepcidin-independent, as it is taken up to the lymphatic circulation through transcellular and M cell-mediated endocytosis routes [9]. This gives Sucrosomial® iron the ability to increase Hb levels, even in the presence of inflammation, as demonstrated in cardiac surgery [25, 26] and in patients with chronic kidney disease [27], cancer [28] or inflammatory bowel disease [29, 30]. Notably, the efficacy of Sucrosomial® iron is observed at much lower dose and with fewer gastrointestinal adverse events compared to oral iron sulfate.

Furthermore, the difference in Hb increment between the study groups at T10 also suggests a correlation between the dose used (60 mg/day vs. 30 mg/day) and the speed of Hb recovery. As endogenous erythropoietin production is higher at low Hb concentrations, a higher initial oral Sucrosomial® iron dose may result in a faster recovery from PPIDA. Subsequently, the dose could be reduced to ensure adequate iron supply to the bone marrow. In this regard, Giordano et al. (2021) [14] recently demonstrated the efficacy and tolerability of high-dose oral Sucrosomial® iron (120 mg elemental iron/day) for 4 weeks, compared to intravenous ferric gluconate, in severe IDA patients intolerant/refractory to iron sulfate.

With regards to iron biomarkers, the accurate assessment of the actual effect of Sucrosomial® iron on the improvement of ferritin and TSAT levels was hampered by the influence of inflammation on the levels of these biomarkers at T0 [31]. Therefore, instead of evaluating the differences between T0 and T60, we assessed the percentage of patients presenting with ferritin and TSAT levels above their thresholds for defining ID at T60, when no inflammation is present.

A serum ferritin level < 30 ng/mL is the most sensitive (92%) and specific (98%) cut-off level for the identification of true ID (with or with-out anaemia) and a TSAT < 20% is insufficient to meet the iron requirements for erythropoiesis [32]. In our study, 45% of the patients achieved a TSAT level within the normal range (≥ 20%), and 36% achieved a ferritin level within the normal range (≥ 30 ng/mL) at T60. We used these thresholds in accordance with current guidelines for the diagnosis of ID [1, 5, 33], although more conservative thresholds are generally used in women (12–18 ng/mL for ferritin, and 15% for TSAT).

Moreover, as mean ferritin levels remained largely unchanged at T60, it can be inferred that the absorbed iron was mainly utilized for erythropoiesis and other physiological functions, such as energy production, rather than for building up iron stores. Therefore, Sucrosomial® iron replacement should be extended, at least, up to the third month of the postpartum period to replenish iron stores, as recommended in guidelines [5].

This research has several limitations. Firstly, it is a study pilot, and there is no control group of untreated patients. The lack of a control group is acknowledged as a study limitation, and it represents a shortcoming. However, it is also noteworthy that leaving a proportion of PPIDA untreated is not appropriate as it poses a risk to both the mother's health and the newborn's care. Therefore, it was decided that all participants would receive treatment. Secondly, compared to previous studies, our study had a limited follow-up time and a relatively small sample size, which may result in diminished predictive capacity, limited value for demographic variables, and lower implication in conclusions. We acknowledge the value of undertaking broader and longer trials on various cohorts of postpartum anemia women to validate our results. However, the demonstrated effectiveness of Sucrosomial® iron and the absence of associated gastrointestinal side effect in a difficult-to-treat population may have significance in clinical practice [30, 33, 34]. In particular, commitment to therapy remains a core determinant of positive health outcomes for postpartum women in need of iron supplementation. We assume that Sucrosomial® iron has the potential to enhance adherence to iron supplementation in postpartum women.

## Conclusion

This pilot study provides preliminary evidence supporting the potential effectiveness of Sucrosomial® iron in the treatment of mild and/or moderate postpartum IDA in women. The results indicate a significant increase in hemoglobin levels in both groups, observed as early as 10 days after treatment initiation. While the study has limitations due to the absence of a control group and a limited sample size, these findings warrant further investigation in larger and longer-term trials. Sucrosomial® iron may represent a promising alternative for the treatment of PPIDA, and future studies should aim to compare its efficacy and safety with standard iron supplementation protocols.

## Data Availability

The datasets used and/or analyzed during the current study are available from the corresponding author on reasonable request.

## Abbreviations

BMI: Body Mass Index
CRP: C-Reactive Protein
Hb: Hemoglobin
HIV: Human Immunodeficiency Virus
ID: Iron Deficiency
IDA: Iron Deficiency Anaemia
PPH: Postpartum Hemorrhage
RBC: Red Blood Cells
SBP: Systolic Blood Pressure
SD: Standard Deviation
sTfR: Soluble Transferrin
TSAT: Transferrin Saturation
WHO: World Health Organization

## References

[1] Muñoz M, Peña-Rosas JP, Robinson S, Milman N, Holzgreve W, Breymann C, Goffinet F, Nizard J, Christory F, Samama CM, Hardy JF (2018). Patient blood management in obstetrics: management of anaemia and haematinic deficiencies in pregnancy and in the post-partum period: NATA consensus statement. Tranfus Med.

[2] Bergmann RL, Richter R, Bergmann KE, Dudenhausen JW (2010). Prevalence and risk factors for early postpartum anaemia. Eur J Obstet Gynecol Reprod Biol.

[3] Milman N (2011). Postpartum anaemia I: definition, prevalence, causes and consequences. Ann Hematol.

[4] Milman N (2012). Postpartum anaemia II: prevention and treatment. Ann Hematol.

[5] Pavord S, Daru J, Prasannan N, Robinson S, Stanworth S, Girling J (2020). BSH Committee.UK guidelines on the management of iron deficiency in pregnancy. Br J Haematol..

[6] Pratt JJ, Khan KS (2016). Non-anaemic iron deficiency - a disease looking for recognition of diagnosis: a systematic review. Eur J Haematol.

[7] Muñoz M, Stensballe J, Ducloy-Bouthors A-S, Bonnet M-P, De Robertis E, Fornet I, Goffinet F, Hofer S, Holzgreve W, Manrique S, Nizard J, Christory F, Samama CM, Hardy JF (2019). Patient blood management in obstetrics: prevention and treatment of postpartum haemorrhage. A NATA Consensus Statement Blood Transfus.

[8] Muñoz M, Gómez-Ramírez S, Besser M, Pavía J, Gomollón F, Liumbruno GM, Bhandari S, Cladellas M, Shander A, Auerbach M (2017). Current misconceptions in diagnosis and management of iron deficiency. Blood Transfus.

[9] Gómez-Ramírez S, Brilli E, Tarantino G, Muñoz M (2018). Sucrosomial® Iron: A New Generation Iron for Improving Oral Supplementation. Pharmaceuticals (Basel).

[10] Parisi F, Berti C, Mandò C, Martinelli A, Mazzali C, Cetin I (2017). Effects of different regimens of iron prophylaxis on maternal iron status and pregnancy outcome: a randomized control trial. J Matern Fetal Neonatal Med.

[11] Berardi S, Foltran L, Pascoli I, Pepe A, Salmeri M.G, Busato E (2016). Efficacy of oral Sucrosomial® iron in puerperium anemia. Exp. Rev. Hematol.

[12] Breymann C, Auerbach M (2017). Iron deficiency in gynecology and obstetrics: clinical implications and management. Hematology Am Soc Hematol Educ Program.

[13] R Core Team. R: A language and environment for statistical computing. R Foundation for Statistical Computing, Vienna, Austria. 2021. URL https://www.R-project.org/.

[14] Giordano G, Napolitano M, Di Battista V, Lucchesi A (2021). Oral high-dose sucrosomial iron vs intravenous iron in sideropenic anemia patients intolerant/refractory to iron sulfate: a multicentric randomized study. Ann Hematol.

[15] Peña-Rosas JP, De-Regil LM, Dowswell T, Viteri FE (2015). Daily oral iron supplementation during pregnancy. Cochrane Database Syst Rev.

[16] Seid MH, Derman RJ, Baker JB, Banach W, Goldberg C, Rogers R (2008). Ferric carboxymaltose injection in the treatment of postpartum iron deficiency anemia: a randomized controlled clinical trial. Am J Obstet Gynecol.

[17] Van Wyck DB, Martens MG, Seid MH, Baker JB, Mangione A (2007). Intravenous ferric carboxymaltose compared with oral iron in the treatment of postpartum anemia: a randomized controlled trial. Obstet Gynecol.

[18] Breymann C, Gliga F, Bejenariu C, Strizhova N (2008). Comparative efficacy and safety of intravenous ferric carboxymaltose in the treatment of postpartum iron deficiency anemia. Int J Gynaecol Obstet.

[19] Froessler B, Cocchiaro C, Saadat-Gilani K, Hodyl N, Dekker G (2013). Intravenous iron sucrose versus oral iron ferrous sulfate for antenatal and postpartum iron deficiency anemia: a randomized trial. J Matern Fetal Neonatal Med.

[20] Lebrecht A, Häberlin F, Eberhard J (1995). Anemia in puerperium; parenteral iron substitution renders erythropoietin therapy dispensable. Geburtshilfe Frauenheilkd.

[21] Breymann C, Zimmermann R, Huch R, Huch A (1996). Use of recombinant human erythropoietin in combination with parenteral iron in the treatment of postpartum anaemia. Eur J Clin Invest.

[22] Breymann C, Richter C, Huch R, Huch A (2000). Effectiveness of recombinant erythropoietin and iron sucrose vs. iron therapy only, in patients with postpartum anaemia and blunted erythropoiesis. Eur J Clin Invest.

[23] Gredilla E, Gimeno M, Canser E, Martínez B, Pérez Ferrer A, Gilsanz F (2006). Tratamiento de la anemia en el postparto y en el postoperatorio inmediato de cirugía ginecológica, con hierro intravenoso Postpartum and early postoperative anemia after gynecological surgery: treatment with intravenous iron. Rev Esp Anestesiol Reanim.

[24] Karavidas A, Troganis E, Lazaros G, Balta D, Karavidas IN, Polyzogopoulou E, Parissis J, Farmakis D (2021). Oral sucrosomial iron improves exercise capacity and quality of life in heart failure with reduced ejection fraction and iron deficiency: a non-randomized, open-label, proof-of-concept study. Eur J Heart Fail.

[25] Pierelli L, De Rosa A, Falco M, Papi E, Rondinelli MB, Turani F, Weltert L (2021). Preoperative Sucrosomial Iron Supplementation Increases Haemoglobin and Reduces Transfusion Requirements in Elective Heart Surgery Patients: A Prospective Randomized Study. Surg Technol Int.

[26] Venturini E, Iannuzzo G, Lorenzo DI, A, Cuomo G, D'Angelo A, Merone P, Cudemo G, Pacileo M, D'Andrea A, Vigorito C, Giallauria F.  (2022). Short-term treatment of iron deficiency anemia after cardiac surgery. Int J Cardiol Heart Vasc.

[27] Pisani A, Riccio E, Sabbatini M, Andreucci M, Del Rio A, Visciano B (2015). Effect of oral liposomal iron versus intravenous iron for treatment of iron deficiency anaemia in CKD patients: A randomized trial. Nephrol Dial Transplant.

[28] Mafodda A, Giuffrida D, Prestifilippo A, Azzarello D, Giannicola R, Mare M, Maisano R (2017). Oral sucrosomial iron versus intravenous iron in anemic cancer patients without iron deficiency receiving darbepoetin alfa: A pilot study. Support Care Cancer.

[29] Bertani L, Tricò D, Zanzi F, Baiano Svizzero G, Coppini F, de Bortoli N, Bellini M, Antonioli L, Blandizzi C, Marchi S (2021). Oral Sucrosomial Iron Is as Effective as Intravenous Ferric Carboxy-Maltose in Treating Anemia in Patients with Ulcerative Colitis. Nutrients.

[30] Abbati G, Incerti F, Boarini C, Pileri F, Bocchi D, Ventura P, Buzzetti E, Pietrangelo A (2019). Safety and efficacy of sucrosomial iron in inflammatory bowel disease patients with iron deficiency anaemia. Intern Emerg Med.

[31] Muñoz M, García-Erce JA, Remacha AF (2011). Disorders of iron metabolism. Part II: Iron deficiency and iron overload. J Clin Pathol.

[32] Camaschella C (2015). Iron-deficiency anemia. N Engl J Med.

[33] Kumar A, Sharma E, Marley A, Samaan MA, Brookes MJ (2022). Iron deficiency anaemia: pathophysiology, assessment, practical management. BMJ Open Gastroenterol.

[34] Elli L, Ferretti F, Branchi F, Tomba C, Lombardo V, Scricciolo A, Doneda L, Roncoroni L (2018). Sucrosomial iron supplementation in anemic patients with celiac disease not tolerating oral ferrous sulfate: a prospective study. Nutrients.

